# Integrated multi-omics analysis reveals that SMARCAL1 serves as an immune related prognostic marker and promotes liver cancer growth

**DOI:** 10.1097/JS9.0000000000004567

**Published:** 2025-12-17

**Authors:** Tingting Huang, Meng Tang, Qiulin Tang, Di Ye, Jiayu Wang, Yichun Duan, Chenliang Zhang, Feng Bi

**Affiliations:** a Division of Abdominal Cancer, Department of Medical Oncology, Cancer Center and Laboratory of Molecular Targeted Therapy in Oncology, West China Hospital, Sichuan University, Chengdu, China; b Department of Laboratory Medicine, West China Hospital, Sichuan University, Chengdu, Sichuan, China; c School of Medicine and Health, Harbin Institute of Technology, Harbin, China

**Keywords:** Hippo-YAP pathway, immune infiltration, liver cancer, proliferation, SMARCAL1

## Abstract

**Objective:**

To illustrate the potential role of SMARCAL1 in malignancy, immune regulation, and related pathway in liver cancer.

**Methods:**

In our study, we examined the expression patterns, clinical features, and survival analysis from The Cancer Genome Atlas and GEO datasets. Immunedeconv package was employed for evaluating the association between the immune infiltration components and SMARCAL1 expression. Knockdown experiment was performed to examine the impacts of SMARCAL1 on cell viability, apoptosis, and classical Hippo-YAP pathway. Meanwhile, the association between SMARCAL1 expression and drug sensitivity was constructed through CTRP, PRISM, and GDSC databases. Finally, we also discovered novel inhibitors of SMARCAL1 activity against liver cancer and YAP activity through virtual screening and western blot assay.

**Results:**

SMARCAL1 exhibited increased expression in liver cancer (LIHC) and was linked to unfavorable outcomes for patients. SMARCAL1 expression was associated with specific clinical features, drug sensitivity, and immune microenvironment characteristics. Additionally, SMARCAL1 was closely related to the activity of Hippo-YAP pathway. Finally, deferoxamine mesylate and its parent compound deferoxamine were identified as candidate inhibitors of SMARCAL1 activity, showing significant ability to inhibit proliferation and the downstream protein YAP1.

**Conclusion:**

Targeting SMARCAL1 could be a promising strategy for liver cancer treatment.

## Introduction

Liver cancer (LC) persists as one of the most common and fatal malignant tumors worldwide^[1]^. Despite being a primary therapeutic intervention, surgical resection demonstrates limited applicability during initial diagnosis, with numerous patients presenting contraindications for operative procedures^[2]^. Contemporary management strategies emphasize molecularly targeted agents and immunomodulatory approaches as pivotal components of oncologic care^[3]^. Nevertheless, monotherapy regimens often yield suboptimal therapeutic responses, prompting increased investigation into synergistic combination protocols^[4]^. This imperative clinical scenario underscores the critical need for identifying novel molecular targets to enhance therapeutic precision in hepatic oncology.

**HIGHLIGHTS**

- Elevated SMARCAL1 expression was statistically associated with unfavorable prognosis in liver cancer.
- SMARCAL1 serves as an immune related prognostic marker and regulates the activity of Hippo/YAP signaling in liver cancer.

SMARCAL1, an SNF2 family ATP-dependent chromatin remodeler, plays a role in multiple cellular processes^[5]^, including gene transcription, DNA damage repair^[6]^, DNA recombination, telomere maintenance, and cell cycle regulation^[7]^. A study in 2023 showed that SMARCAL1 deficiency is permissive to alternative lengthening of telomeres and promotes gliomagenesis^[8]^. Moreover, SMARCAL1 loss was also found in giant cell glioblastoma^[9]^. Prior studies have established that alterations in the SMARCAL1 gene play a key role in the development of Schimke immuno-osseous dysplasia, a rare autosomal recessive condition marked by impaired T-cell immunity and stunted growth^[10]^. More importantly, a significant study in 2024 has demonstrated SMARCAL1 as a key dual regulator in curbing endogenous DNA damage to suppress CGAS-STING-mediated immune responses in cancer, thereby promoting tumor immune evasion^[11]^. Additionally, SMARCAL1 was found to be an oncogene in small cell lung cancer (SCLC)^[12]^, and pixantrone possessed therapeutic potential in SCLC as a SMARCAL1 inhibitor^[13]^. These findings underscore the significant potential of SMARCAL1 as an innovative therapeutic target; however, the functional characteristics of SMARCAL1 in HCC remain to be elucidated.

In this study, we systematically demonstrated the SMARCAL1 profiles, including mRNA expression, stages, survival prognosis, immune infiltration, and functional enrichment pathway through multi-omics database integration. *In vitro* experiments, we confirmed that SAMRCAL1 not only promoted the proliferation and inhibited apoptosis of HCC cells but also regulated the activity of Hippo-YAP, the classic tumor suppressor pathway in HCC. Furthermore, we obtained promising SMARCAL1 inhibitors by virtual screening, which also inhibited the proliferation and YAP1 expression in HCC, demonstrating the regulatory necessity of SMARCAL1 in Hippo pathway. Therefore, SMARCAL1 could serve as an oncogene through enhanced proliferation, suppressed apoptosis, and upregulated expression significantly correlated with reduced patient survival, thereby systematically elucidating its pivotal molecular mechanism in HCC pathogenesis.

## Method and material

### Data extraction and differential expression of SMARCAL1

The Cancer Genome Atlas (TCGA)-Liver Hepatocellular Carcinoma (LIHC) cohort data were downloaded from TCGA database, and the RNAseq and corresponding clinical data of patients were extracted. A total of 374 LIHC patients were finally included in this study. Meanwhile, the clinicopathological data includes pathological stage, TNM stage, tumor status, age, gender, residual tumor, histological grade, alpha-fetoprotein, albumin, prothrombin time, vascular invasion, fibrosis Ishak score, and the survival status [overall survival (OS), disease-specific survival (DSS), and progression-free interval (PFI)]. Transcriptomic profiles from the TCGA-LIHC dataset were processed through the Limma package, applying stringent differential expression thresholds of absolute log2 fold change >1 combined with adjusted *P* < 0.05. GEO datasets (GEO29721, GSE67764, GSE112790, GSE6764, GSE17548, and GSE63067) including hepatitis, liver cirrhosis, and tumor were also extracted in this study, normalized by log2 transformation and standardized by the preprocessCore package of R^[14]^. Finally, protein differences of SMARCAL1 in liver cancer and the control group were measured by the Human Protein Atlas (HPA) database. The work has been reported in line with the TITAN criteria and REMARK criteria^[15,16]^.

### Survival analysis and univariate/multivariate Cox regression

Using Kaplan-Meier survival analysis and Cox regression analysis, we explored the prognostic value of SMARCAL1 in liver cancer. The survival package was used to conduct a proportional hazards hypothesis test, followed by fit survival regression and Cox regression analysis. The 95% confidence interval (CI) represents the range of uncertainty surrounding the hazard ratio (HR). The median time reflects the duration corresponding to a 50% survival rate in different groups, often referred to as the median survival time, measured in months or years. When the samples in the univariate analysis meet the set *P*-value threshold (*P* < 0.1), multivariate Cox analysis will be conducted. Ultimately, the results were visualized using the survminer and ggplot2 packages.

### Characteristics of SMARCAL1-related immune infiltration in liver cancer

Single-cell profiling of SMARCAL1 distribution across immune cell subtypes was performed using .H5-formatted single-cell repositories acquired from the TISCH database. Data processing pipelines integrated the MAESTRO analytical framework with Seurat-based dimensional reduction workflows, followed by cellular subpopulation reclassification via t-Distributed Stochastic Neighbor Embedding (t-SNE) nonlinear dimensionality reduction^[17]^. For comprehensive immune landscape quantification, a multi-algorithmic immunedeconv toolkit incorporating TIMER, xCell, MCP-counter, CIBERSORT, EPIC, and quanTIseq methodologies was implemented, with subsequent network visualization achieved through the ggClusterNet graphical ecosystem^[18,19]^. Furthermore, the “Immune Association” module of Timer database was utilized for exploring the relationship between immune infiltrates and clinical outcome in liver cancer^[20]^.

### Function annotation and drug analysis

Here, chemotherapeutic susceptibility and resistance for SMARCAL1-associated phenotypes in LIHC was performed via the “oncoPredict” package through GDSC, CTRP and PRISM databases. Besides, differential expression profile of SMARCAL1 was then conducted through Limma algorithm (v3.40.2), with a threshold of “Adjusted *P* < 0.05 and log2 (fold change) > 1 or log2 (fold change) < −1” and heatmap is used to visualize the predicted drugs related to SMARCAL1. Use the ClusterProfiler package in R to analyze the GO functions of potential mRNAs and to enrich the KEGG pathways.

### Molecular docking

The crystal structure of SMARCAL1 and the molecular docking between Lenvatinib and Sorafenib were studied. Both the protein and ligand structures were prepared using ds3.0 software, including the addition of hydrogen, the correction of incomplete residues, and the removal of water molecules. CHARMM force field was used to assign electrostatic potential to proteins. Subsequently, molecular docking is performed using the -CDOCKER program^[21]^. The prepared ligand pairs are attached to the protein bag and then classified according to the docking fraction. In protein-protein docking, HDock server (http://hdock.phys.hust.edu.cn/) was used to determine the protein complexes between possible binding sites^[22]^. All compounds were constructed using the Sketch module in SYBIL-X 2.0 (Tripos Inc., St. Louis, MO), and the molecules were optimized using the Gasteicer-Huckel charge optimization method. The receptor YAP (3KYS) was obtained from the Protein Data Bank (PDB) and the SWISS-MODEL server for homology modeling. Additionally, the Natural compounds library MedChemExpress (MCE) containing 4916 compounds was used as the target library to screen candidate compounds of SMARCAL1 for molecular docking. After completion of docking, the results were analyzed using PyMOL software.

### Molecular dynamics simulations

To assess the binding dynamics between potential compounds and target proteins, molecular dynamics (MD) simulations were conducted using the highest-ranking docking poses derived from re-docking analyses. These simulations were executed in a Linux-based system utilizing the Desmond module within the Schrodinger suite, aimed at evaluating the stability of the protein-ligand interactions. The system was hydrated using the simple point charge water model under periodic boundary conditions, incorporating sodium ions to maintain charge neutrality. The OPLS 2005 force field was applied for optimization, with a convergence criterion established at 1.0 kcal/mol/Å^2^ and a maximum interaction threshold set at 2000^[23]^. Energy minimization was performed using the limited memory Broyden-Fletcher-Goldfarb-Shanno (LBFGS) algorithm, and steepest descent was subsequently implemented to achieve a gradient threshold of 25 kcal/mol/Å. Structural stability and simulation equilibrium were evaluated by monitoring the root mean square deviations of the protein backbone, crucial active-site residues, and ligand atoms.

### Cell line and cell culture

SNU387, SNU423, and HEPG2 human liver cancer cells were cultured as previous describe^[24]^. The medium in which they were placed was added with 10% fetal bovine serum (Gibco, USA) and then put into an incubator under the conditions of 37°C and 5% carbon dioxide.

### RNA isolation and quantitative real-time PCR

We used the Trizol reagent (TIANGEN, China) to extract the total RNA from the liver cancer cells. Then, cDNA reverse transcription and qRT-PCR process were conducted following the established protocol. Expression patterns of the target genes were standardized to GAPDH and calculated as ΔCT values. We cataloged the primers contained in this study, as demonstrated in Supplemental Digital Content Table S1, available at: http://links.lww.com/JS9/G498.

### Small interfering RNA and plasmids transfection

To target SMARCAL1 and YAP1, small interfering RNAs (siRNAs) were synthesized by Tsingke Biotech Co., Ltd. in Beijing. Cells were transfected with siRNA/plasmid complexes using Lipofectamine 2000 (Invitrogen), following the manufacturers protocol. After 24-hour post-transfection incubation, the cells were harvested for qRT-PCR analysis. The siRNA sequence included in this research is listed in Supplemental Digital Content Table S2, available at: http://links.lww.com/JS9/G498.

### Cell proliferation and colony formation

The proliferative consequences of SMARCAL1 regulation in liver cancer cells were quantitatively evaluated using the CCK-8 assay. The cells were uniformly planted in 96-well plates with densities not exceeding 50%. After that, 100 µL medium containing 10%CCK8 was incubated in each hole to be tested. The absorbance at wavelength of 450 nm was measured 2 h after adding CCK-8. For colony formation assay, liver cancer cells with or without siSMARCAL1 were seeded in 12-well plates at a certain density of per well. After 5 days, the cells were washed three times with PBS and stained with 0.2% crystal violet. Surviving colonies were defined as those containing more than 50 cells.

### Western blot analysis and flow cytometry

As previously detailed, immunoblotting analysis and immunoprecipitation were carried out^[25]^. Colored gel formulation kit (EC1023-B, Shandong Sparkjade Biotechnology Co., Ltd.) for isolation of protein samples and visualization of detection. The primary antibodies utilized in this study are as follows: SMARCAL1 (1:1000 dilution, Proteintech, China), YAP (1:1000 dilution, Proteintech, China), p-YAP (1:1000 dilution, Abcam, UK), CTGF (1:1000 dilution, Proteintech, China), β-catenin (1:1000 dilution, Abcam, UK), Cyclin D1 (1:1000 dilution, Proteintech, China), and β-actin (1:1000 dilution, Proteintech, China). The Annexin V-FITC Apoptosis detection kit was used for the detection of apoptosis in our study (ABclonal, wuhan, China). Cells were digested and washed twice with PBS and then resuspended in 200 µL binding buffer, and add FITC conjugated annexin V antibody (2 µL) and propidium iodide (2 µL) for 10 min on ice in the dark. Data analysis was performed using flow cytometer (Beckman Coulter).

### Statistical analysis

Statistical evaluations were applied utilizing the designated online database and R package (version 4.4.1), as previously outlined. Survival analysis was performed using Cox regression and Log-rank test. The inter-group comparison of categorical variables was conducted using the chi-square test. The specific statistical methods are as follows: (1) When the data meet the conditions of theoretical frequency all >5 and total sample size ≥40, the chi-square test was used; (2) 5 > theoretical frequency ≥ 1 and total sample size ≥ 40, the continuous correction chi-square test (Yates’ correction) was used; (3) theoretical frequency < 1 or total sample size < 40, the Fisher’s exact test was used. Continuous variables were summarized as mean ± SD, and the intergroup comparison was performed using ANOVA test. This analysis process was accomplished through GraphPad Prism 9.0 software. Statistical significance was marked with *P < 0.05, **P < 0.01, and ***P < 0.001.

## Results

### Deciphering the expression characteristics of SMARCAL1 in liver cancer

Dysregulation of the SWI/SNF complex has long been associated with tumor progression and deterioration, and recently, members of the complex have been reported to be involved in regulating the biological processes of different cancers, such as SMARCA4 in lung cancer and SMARCB1 in renal cancer^[26,27]^. Here, we primarily performed an intersection of SWI/SNF family with HCC prognostic genes and found that 11 family members were found to be associated with the prognosis of HCC (Supplemental Digital Content Figure S1A, available at: http://links.lww.com/JS9/G498). Then, the cox analysis revealed that they might function as independent risk roles in HCC (Supplemental Digital Content Figure S1B, available at: http://links.lww.com/JS9/G498). Meanwhile, we also investigated the differential expression of these 11 genes between HCC tissues and their corresponding adjacent tissues and normal tissues and found that they were all highly expressed in HCC, suggesting that they may act as oncogenes affecting the progression of HCC (Supplemental Digital Content Figure S1C-D, available at: http://links.lww.com/JS9/G498).

**Figure 1.**
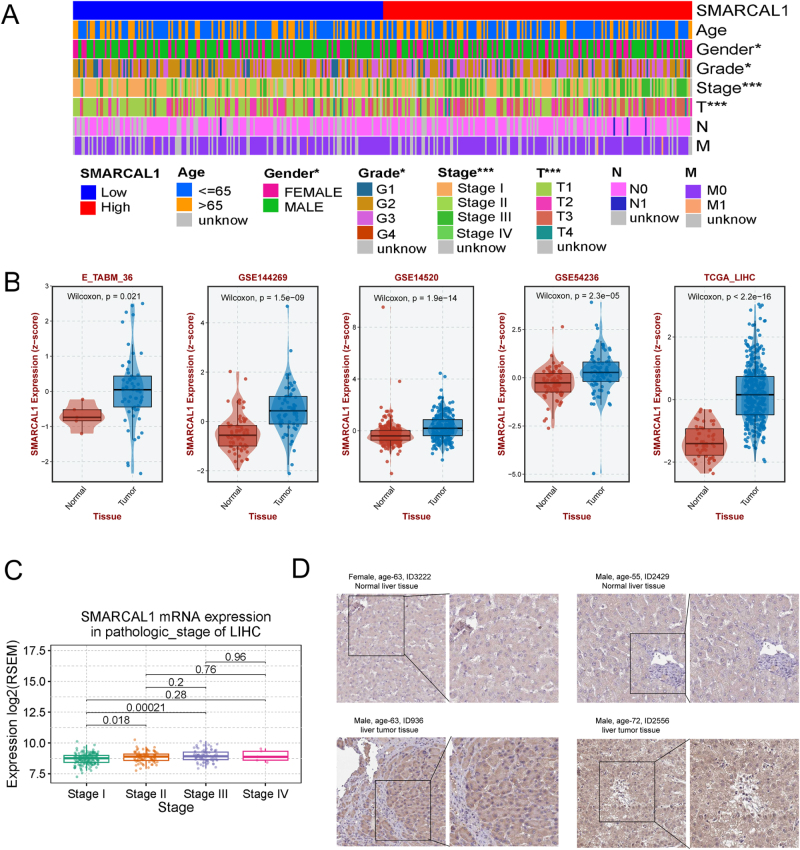
Relationship between SMARCAL1 expression and clinicopathological characteristics in liver cancer. (A) The landscape of clinical feature of HCC patients related to SMARCAL1 in TCGA database. (B) The expression of SMARCAL1 in subgroup with different datasets. (C) The expression of SMARCAL1 in different stages in HCC cohort. (D) Demonstration of SMARCAL1 protein expression in tumor tissue versus non-tumor tissue using the Human Protein Atlas (HPA) database.

SMARCAL1, an important SWI/SNF subunit gene, was previously reported to be a new target for cancer treatment. However, its specific role in liver cancer remains unclear. Therefore, we further evaluated the relationship between SMARCAL1 expression and the clinical phenotypic characteristics of liver cancer, as displayed in Figure 1A. The heatmap illustrated that SMARCAL1 expression was significantly related to gender, tumor grade, and stage of liver cancer. In specific, the detailed baseline data of liver cancer patients obtained from TCGA are shown in Table 1. SMARCAL1 was expressed at a higher level in liver cancer tissues compared to normal tissues from various datasets (E_TABM_36, GSE144269, GSE14520, GSE5423, and TCGA; Fig. 1B). We also analyzed the expression landscapes of SMARCAL1 in different liver diseases: liver cancer, hepatitis, and liver cirrhosis (Supplemental Digital Content Figure S2C-D, available at: http://links.lww.com/JS9/G498). Our study found that SMARCAL1 was significantly highly expressed in liver cancer, while no statistical differences were detected in hepatitis and liver cirrhosis compared with normal tissues, suggesting that SMARCAL1 could be a potential biomarker for diagnosing liver cancer. Further analysis indicated that SMARCAL1 also demonstrated independent statistical significance in the staging of liver cancer, and the result of immunohistochemical staining indicated that the expression of SMARCAL1 was elevated in liver cancer patients, suggesting that it may play a role in promoting carcinogenesis (Fig. 1C-D).

**Table 1:** Correlations of SMARCAL1 expression with various characteristics in liver cancer.

Characteristics	Low expression of SMARCAL1	High expression of SMARCAL1	*P* value
*n*	187	187	
Pathologic stage, *n* (%)			<0.001
Stage I	104 (29.7%)	69 (19.7%)	
Stage II	40 (11.4%)	47 (13.4%)	
Stage III	29 (8.3%)	56 (16%)	
Stage IV	3 (0.9%)	2 (0.6%)	
Pathologic T stage, *n* (%)			0.004
T1	108 (29.1%)	75 (20.2%)	
T2	42 (11.3%)	53 (14.3%)	
T3	31 (8.4%)	49 (13.2%)	
T4	4 (1.1%)	9 (2.4%)	
Pathological N stage, *n* (%)			0.659
N0	124 (48.1%)	130 (50.4%)	
N1	1 (0.4%)	3 (1.2%)	
Pathological M stage, *n* (%)			1.000
M0	131 (48.2%)	137 (50.4%)	
M1	2 (0.7%)	2 (0.7%)	
Tumor status, *n* (%)			0.104
With tumor	70 (19.7%)	83 (23.4%)	
Tumor free	110 (31%)	92 (25.9%)	
Residual tumor, *n* (%)			0.081
R0	172 (49.9%)	155 (44.9%)	
R1	5 (1.4%)	12 (3.5%)	
R2	1 (0.3%)	0 (0%)	
Histologic grade, *n* (%)			0.018
G1	30 (8.1%)	25 (6.8%)	
G2	102 (27.6%)	76 (20.6%)	
G3	49 (13.3%)	75 (20.3%)	
G4	5 (1.4%)	7 (1.9%)	
Alpha-fetoprotein, ng/mL), *n* (%)			<0.001
≤400	131 (46.8%)	84 (30%)	
>400	18 (6.4%)	47 (16.8%)	
Albumin (g/dL), *n* (%)			0.635
<3.5	36 (12%)	33 (11%)	
≥3.5	128 (42.7%)	103 (34.3%)	
Prothrombin time, *n* (%)			0.989
≤4	112 (37.7%)	96 (32.3%)	
>4	48 (16.2%)	41 (13.8%)	
Vascular invasion, *n* (%)			0.130
Yes	51 (16%)	59 (18.6%)	
No	115 (36.2%)	93 (29.2%)	
Fibrosis Ishak score, *n* (%)			0.714
0	43 (20%)	32 (14.9%)	
1/2	17 (7.9%)	14 (6.5%)	
3/4	16 (7.4%)	12 (5.6%)	
5	3 (1.4%)	6 (2.8%)	
6	42 (19.5%)	30 (14%)	
Overall survival event, *n* (%)			0.017
Alive	133 (35.6%)	111 (29.7%)	
Dead	54 (14.4%)	76 (20.3%)	
Disease-specific survival event, *n* (%)			0.231
No	149 (40.7%)	138 (37.7%)	
Yes	35 (9.6%)	44 (12%)	
Progression-free interval event, *n* (%)			0.255
No	101 (27%)	90 (24.1%)	
Yes	86 (23%)	97 (25.9%)	

### Prognostic value of SMARCAL1 in liver cancer

**Figure 2.**
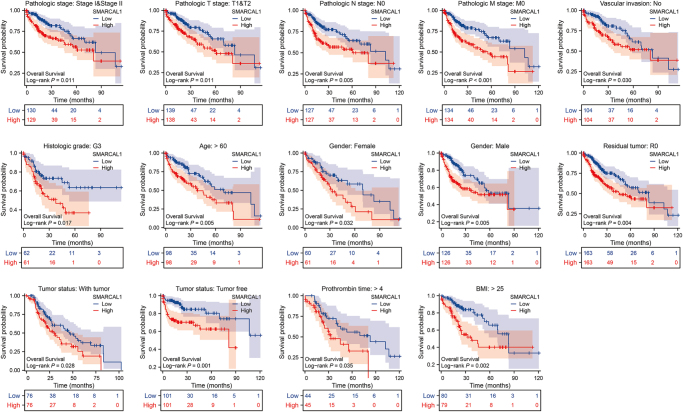
SMARCAL1 makes a negative impact on overall survival (OS) of liver cancer patients categorized into different clinical subtypes, including pathological stage: stage I and stage II, pathological T stage: T1 and T2, pathological N stage: N0, pathological M stage: M0, vascular invasion: No, histological grade: G3, age: >60, gender: male, gender: female, residual tumor: R0, tumor status: with tumor, tumor status: tumor free, prothrombin time: >4, and BMI: >25.

The prognostic significance of various variables, including SMARCAL1 expression, in liver cancer via univariate and multivariate Cox regression analyses was also conducted (Supplemental Digital Content Figure S3, available at: http://links.lww.com/JS9/G498). Univariate analysis showed that high SMARCAL1 expression (HR = 1.908, 95% CI: 1.342–2.711, *P* < 0.001), advanced pathologic stage (Stage III: HR = 2.734, *P* < 0.001; Stage IV: HR = 5.597, *P* = 0.004), and tumor status (with tumor: HR = 2.317, *P* < 0.001) were associated with worse OS. Notably, in multivariate analysis, high SMARCAL1 expression remained an independent prognostic factor for poor OS (HR = 1.758, 95% CI: 1.181–2.618, *P* = 0.005). Furthermore, we analyzed the association between SMARCAL1 expression and OS across different clinical subgroups in liver cancer patients. As shown in Figure 2, high SMARCAL1 expression was related to reduced survival in multiple clinical subgroups, such as pathologic stage I and II group, pathological stage T1 and T2 group, and pathological stage N0 group, pathological stage M0 group, non-vascular invasion group, and histological grade G3 group. These results showed SMARCAL1 could be a high-risk factor for liver cancer patients.

### Drug sensitivity analysis of SMARCAL1 in liver cancer

**Figure 3.**
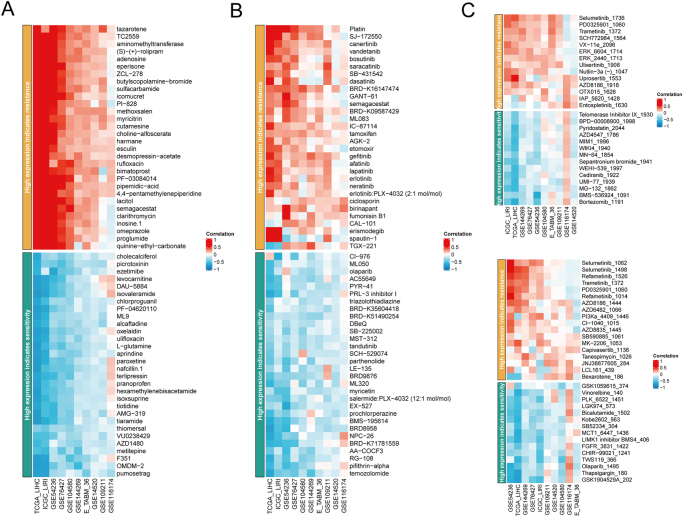
Relationships between SMARCSAL1 expression and clinical drug sensitivity and resistance in HCC through CTRP (A), PRISM (B), and GDSC (C) databases.

Pharmacological treatment favors tumor patients; however, drug resistance always appears^[28]^. For instance, a recent study showed that a large part of advanced HCC patients do not receive long-term benefit from systemic therapy owing to primary and acquired drugs resistance^[29]^. In order to explore the possible application of SMARCAL1 in the personalized treatment of liver cancer, we also evaluated the drug sensitivity and resistance of SMARCAL1 using various pharmacological platforms. Our findings indicated that the high-expression group of SMARCAL1 exhibited greater resistance to Refametinib, Trametinib, ERK inhibitors, Dasatinib, and Gefitinib, among others. Conversely, this group demonstrated increased sensitivity to cholecalciferol, olaparib, PRL-3 inhibitor, bicalutamide, and so on, which provides a reliable idea for future precise drug treatment of liver cancer (Fig. 3). Besides, anti-angiogenesis targeting drugs are the classic first-line drugs for liver cancer treatment, we further detected the MD interaction between SMARCAL1 and Sorafenib/Lenvatinib by molecular stocking, as shown in Supplemental Digital Content Figure S4A-B, available at: http://links.lww.com/JS9/G498. Sorafenib forms a hydrogen bond with 591, 604, 629, and 620 to form Piper bonds, as well as 623 to form additional halogen bonds with Fluorine on Sorafenib, with a binding energy of −9.40 kcal/mol. Lenvatinib interacts with 604, 623, and 601 to form hydrogen bonds, and 605 and 591 to form π-π interactions, with a binding energy of −8.50 kcal/mol. As can be seen from the cavity diagram, Sorafenib has better binding coordination with the target than Lenvatinib. In addition, further MD studies showed that both Lenvatinib and Sorafenib tended to flatten out at 25 ns, and it could be seen in Supplemental Digital Content Figure S4C-D, available at: http://links.lww.com/JS9/G498 that Sorafenib became more stable after 70 ns. Taken together, these studies suggest that SMARCAL1 may be a potential drug target, especially sorafenib, for liver cancer treatment.

### Relationship between SMARCAL1 expression and tumor immune microenvironment

**Figure 4.**
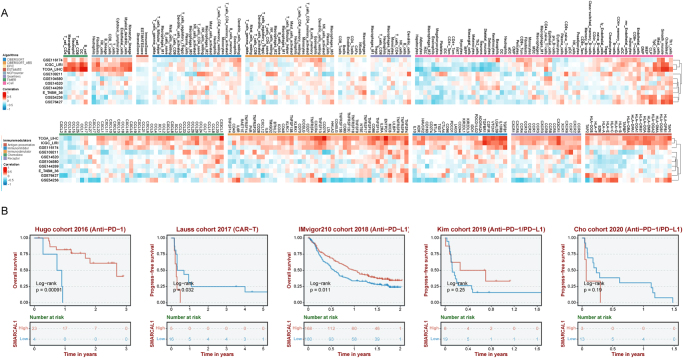
(A) Heatmap illustrating the association between SMARCAL1 expression and immune components including immune infiltration cells and immunomodulators through TCGA and GEO datasets. (B) Survival analysis of SMARCAL1 expression in immune therapy related datasets.

Tumor immune microenvironment (TME) has been proven to be strongly linked to tumor development, recurrence, and metastasis^[30,31]^. Recent research indicates that the condition of tumor-infiltrating immune cells within the TME significantly impacts the effectiveness of immunotherapy^[32]^. Therefore, we investigated the connection between SMARCAL1 expression and immune factors in liver cancer, seeking to further elaborate the function of SMARCAL1 within the immune microenvironment of liver cancer patients. We primarily assessed the association between SMARCAL1 expression and immune cells/immunomodulators and found that SMARCAL1 exerted significant heterogeneity in the patient specimens obtained from different datasets (Fig. 4A). Patients with SMARCAL1-high/CD4 memory resting T cells-low and SMARCAL1-high/CD8 central memory T cells-low exhibited significantly unfavorable survival outcomes. Moreover, patients with SMARCAL1-high combined M2 macrophages-high showed shorter survival time than patients with SMARCAL1-high/M2 macrophages-low, which indicated that targeting SMARCAL1 may improve the complex immune microenvironment of liver cancer patients to make them more sensitive to immunosuppressants (Supplemental Digital Content Figure S5A, available at: http://links.lww.com/JS9/G498).

Then, we conducted a survival analysis on immune therapy datasets, revealing that high expression levels of SMARCAL1 were associated with improved prognosis in the Hugo cohort 2016 (Anti-PD-1) and the IMvigor210 cohort 2018 (Anti-PD-L1). Conversely, elevated SMARCAL1 expression correlates with poorer outcomes in the Lauss cohort 2017 (CAR-T; Fig. 4B). Surprisingly, we also summarized the types of cancer commonly treated with immunosuppressants in clinical practice, including bladder cancer, esophageal cancer, glioblastoma, head and neck squamous cell carcinoma, melanoma, liver cancer, non-SCLC, and urothelial carcinoma. We analyzed the survival outcomes following the administration of immunosuppressants such as PD-1 treatment, PD-L1 treatment, and CTLA-4 treatment. The results indicated that patients in the SMARCAL1-high group exhibited longer survival rates after receiving PD-1 and CTLA-4 treatments. Consequently, targeting SMARCAL1 within the immune microenvironment of liver cancer was worthy of careful exploration (Supplemental Digital Content Figure S5B, available at: http://links.lww.com/JS9/G498).

**Figure 5.**
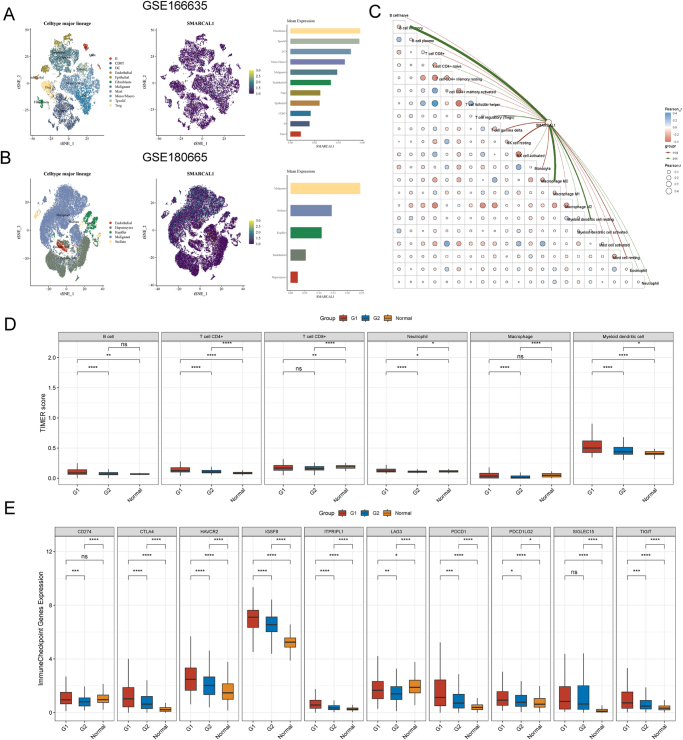
Correlation between SMARCAL1 and immune infiltration in liver cancer. (A and B) The t-SNE plot and bar chart of single-cell clustering illustrating the expression distribution of SMARCAL1 in different cells from GSE166635 and GSE180665 database. Different colors represent different types of cells. The darker the color, the lower the expression of SMARCAL1 in the cell, and the brighter the color, the higher the expression of SMARCAL1 in the cell. (C and D) Correlation between SMARCAL1 and immune cells infiltration in liver cancer via Timer algorithm. (E) Differential analysis of immune checkpoint genes expression and SMARCAL1 expression in liver cancer. G1 in red represents high expression of SMARCAL1, and G2 in blue represents low expression of SMARCAL1. The significance of two groups of samples is evaluated by the Wilcoxon rank-sum test. *P < 0.05, **P < 0.01, and ***P < 0.001.

We also assessed the distribution of SMARCAL1 in various immune-infiltration cells through single cell database and found that SMARCAL1 was most richly expressed in fibroblasts and malignant (Fig. 5A-C). Then, the relation between SMARCAL1 expression and specific immune cells was evaluated via TIMER score, and the results showed that SMARCAL-high group has higher immune score in B cell, CD4 + T cell, Neutrophill, Macrophage, and Myeloid dendritic cell compared to SMARCAL1-low group (Fig. 5D). Furthermore, we also found that upregulated SMARCAL1 expression was related to higher expression level of CD274, CTLA4, HAVCR2, IGSF8, ITPRIPL1, LAG3, PDCD1, PDCD1LG2, and TIGIT (Fig. 5E). These results suggested that SMARCAL1 could be a highly effective immune effector to target liver cancer immunotherapy.

### Correlation of SMARCAL1 expression with biological phenotypes in liver cancer

**Figure 6.**
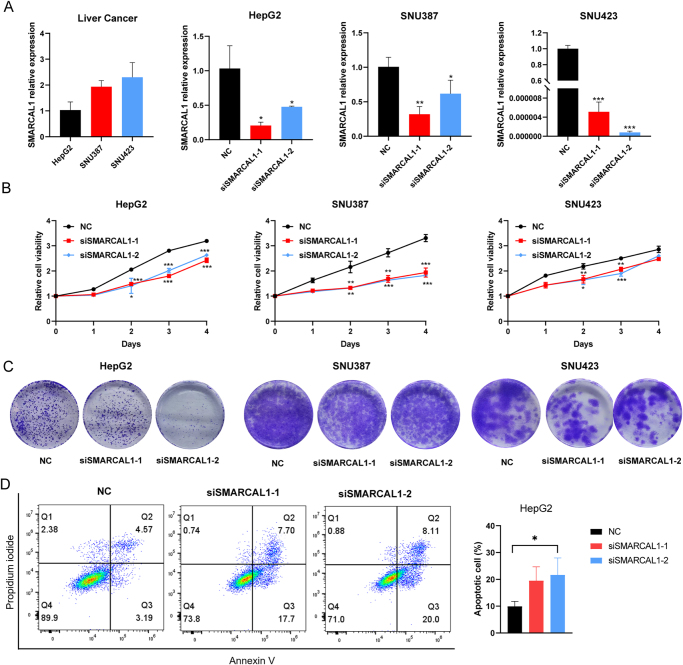
SMARCAL1 promotes the proliferation and inhibits apoptosis of liver cancer cells. (A) qRT-PCR experiment illustrating SMARCAL1 expression of three liver cancer cell lines (HepG2, SNU387, and SNU423) and the knockdown efficiency of SMARCAL1 in the above cell lines. (B) CCK-8 assay showed that cell proliferation is significantly inhibited in HepG2, SNU387, and SNU423 cells at 1, 2, 3, and 4 days post-SMARCAL1 knockdown. (C) Colony formation assay indicated a significant reduction in colony formation ability in these three cell lines following SMARCAL1 knockdown. (D) Flow cytometry detected a significant rising in apoptosis in HepG2 cell following SMARCAL1 knockdown. *P < 0.05, **P < 0.01, and ***P < 0.001.

We also tried to explore the proliferation effect of SMARCAL1 on liver cancer cell lines. We primarily examined the expression level of SMARCAL1 in three liver cancer lines (HepG2, SNU387, and SNU423) and verified the knockdown efficiency of SMARCAL1 in the above three cell lines (Fig. 6A). The CCK-8 assay demonstrated a significant decline in the proliferation rate following the knockdown of SMARCAL1. Additionally, colony formation assays painted a similar picture, with the si-SMARCAL1 group producing far fewer colonies compared to the control group, highlighting the impact of SMARCAL1 depletion on cellular growth (Fig. 6B**-C**). Besides, we also found that reducing SMARCAL1 expression could promote liver cancer apoptosis (Fig. 6D), which indicated that SMARCAL1 may serve as a valuable prognostic biomarker in liver cancer, warranting further research to explore its potential significance in this context.

### Functional enrichment analysis of SMARCAL1 in tumors

**Figure 7.**
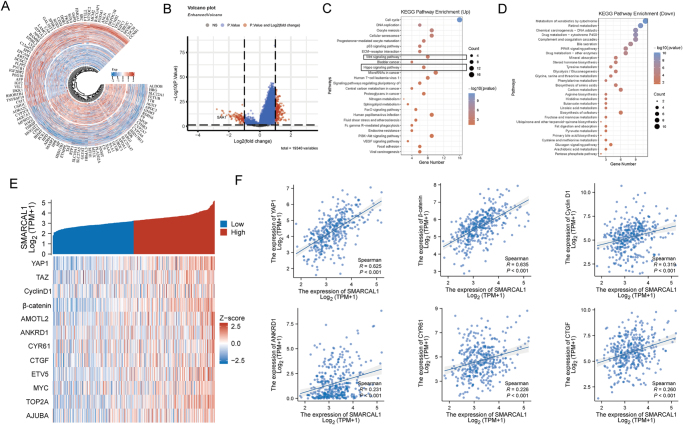
Functional enrichment analysis of SMARCAL1 in HCC. (A) Circos graph of SMARCAL1 co-expression genes. (B) Volcano map representation the SMARCAL1-related genes. (C and D) KEGG enrichment analysis showing SMARCAL1-related pathway. (E and F) Heatmap and scatter diagram illustrated that the association between SMARCAL1 expression and representative biomarkers of Hippo-YAP pathway, such as YAP1, β-catenin, Cyclin D1, ANKRD1, CYR61, and CTGF.

Aiming for further elucidating the function of SMARCAL1 in HCC, we explored the SMARCAL1 co-expression genes, and the results showed that the genes positively associated with SMARCAL1 included CDC20, TPX2, UBE2C, TOP2A, CCNB2, etc., while ALDOB, HRG, SLC22A1, and FETUB were negatively correlated with SMARCAL1 (Fig. 7A). Then, we displayed SMARCAL1-related genes via volcanic map (Fig. 7B). KEGG enrichment pathway illustrated that SMARCAL1 was involved in the regulation of Hippo-Yap pathway in liver cancer (Fig. 7C-D). Therefore, we also evaluated the relation between SMARCAL1 expression and Hippo pathway associated biomarker (including YAP1, β-catenin, Cyclin D1, ANKRD1, CYR61, CTGF, etc.) and indicated that SMARCAL1 was positively relation with the expression of Hippo pathway related markers (Fig. 7E-F).

**Figure 8.**
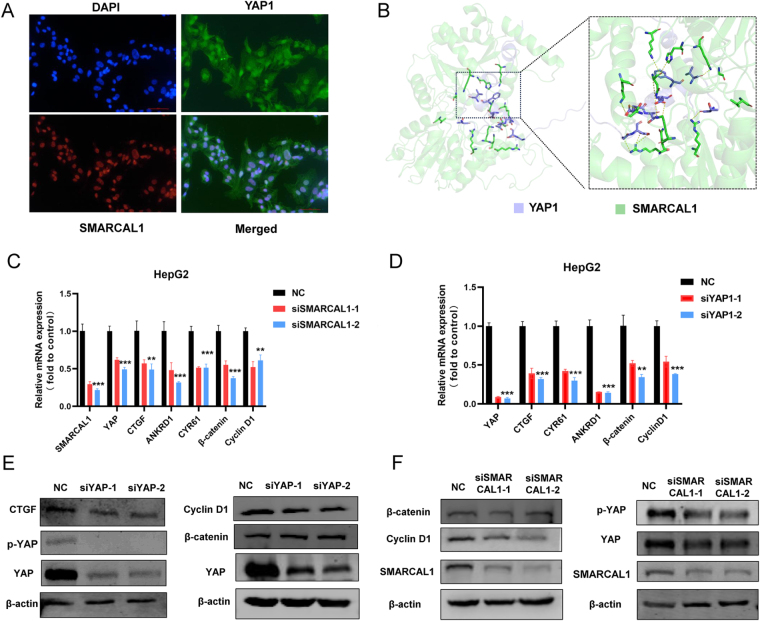
SMARCAL1 was involved in the regulation of Hippo-YAP pathway. (A) The immunofluorescence images illustrating the location of SMARCAL1/YAP1 proteins. Blue represents DAPI, red represents SMARCAL1, and green represents YAP1. (B) Molecular docking revealing the interaction between SMARCAL1 and YAP1. Light purple represents YAP1, and green represents SMARCAL1. (C and D) qRT-PCR experiment illustrated the RNA level of the Hippo pathway effectors (YAP, CTGF, ANKRD1, CYR61, beta-catenin, and Cyclin D1) under the condition of SMARCAL1/YAP knockdown. (E) Western blot detecting the expression on YAP, p-YAP, CTGF, CyclinD1, and β-catenin, in HepG2 cell cultured with the negative control or siYAP. (F) Western blot detecting the expression on YAP, p-YAP, CyclinD1, β-catenin, and SMARCAL1 in HepG2 cell cultured with the negative control or siSMARCAL1.

Then, we investigated the sublocation of SMARCAL1 in HepG2 cell and found that SMARCAL1 was mainly enriched in the nucleus and partially overlapped with the intranuclear portion of YAP1 (Fig. 8A). To further explore the potential regulatory role of SMARCAL1 in Hippo-YAP signaling pathway, we conducted a protein-protein docking analysis of SMARCAL1 and YAP1 (PDB ID: 3KYS), indicating that a stable complex could be formed between the two, with the interaction interface involving multiple key residues from both proteins (Fig. 8B). Specifically, hydrogen bonds were formed between Glu62 of SMARCAL1 and Arg561 of YAP1, and between Asp556 of SMARCAL1 and Lys555 of YAP1; meanwhile, significant hydrophobic interactions were observed between Val72 (SMARCAL1) and Leu725 (YAP1), as well as between Thr77 (SMARCAL1) and His733 (YAP1). Notably, the aromatic ring of His733 on YAP1 might engage in π-π stacking or hydrophobic packing with neighboring residues of SMARCAL1, further stabilizing the complex structure.

Inhibition of YAP activity leads to decreased expression of targeted transcription factors (ANKRD1, CTGF, CYR61, etc.) in the nucleus. Our findings from quantitative real-time PCR also revealed a notable decrease in the levels of YAP, along with a reduction in its target genes ANKRD1, CYR61, CTGF, β-catenin, and Cyclin D1 after SMARCAL1 was inhibited (Fig. 8C-D). Additionally, we also observed YAP protein level decreased, accompanied by the downregulation of p-YAP, and Cyclin D1 under the situation of SMARCAL1 inhibition or YAP knockdown (Fig. 8E-F). Given that YAP over-activation was an important signal for the progression of HCC, the above results indicated that targeting SMARCAL1 could be a promising anti-liver cancer strategy.

### Screening for potential small molecule inhibitors of SMARCAL1

**Figure 9.**
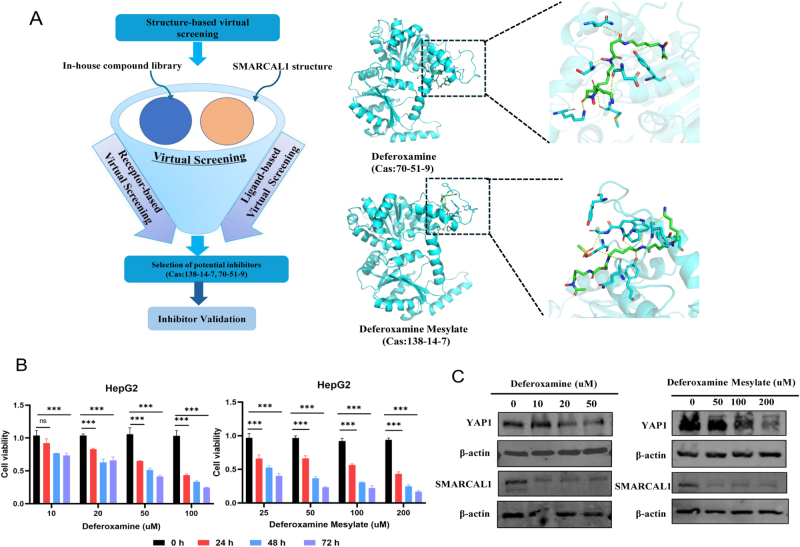
Screening potential small molecule inhibitors of SMARCAL1. (A) Flowchart of virtual screening and molecular docking visualization of candidate ligands and their predicted binding conformations. (B) CCK-8 assay showing that the proliferative ability of HepG2 cell treated with Deferoxamine or Deferoxamine Mesylate for 24 h, 48 h, and 96 h. (C) Effects of SMARCAL1 inhibitors on its downstream signaling protein YAP1. Immunoblotting analysis of YAP1 protein in HepG2 cell treated with Deferoxamine or Deferoxamine Mesylate for 24 h.

To screen for potential small molecule inhibitors of SMARCAL1, we performed a virtual screening of a natural product library containing 4916 compounds. Among the top ranked hits, deferoxamine mesylate and its parent compound deferoxamine showed the strongest binding affinity and stably bound within the putative catalytic pocket of SMARCAL1. As shown in Figure 9A, both of them achieve high affinity binding by forming multiple hydrogen bonds with key residues Asp623, Ser625, and Lys849 and establishing extensive van der Waals interactions with Met584, Tyr591, and Asn629. Notably, deferoxamine mesylate exhibits a slightly better binding conformation than deferoxamine due to its methanesulfonic acid group, suggesting that it has stronger steric and electrostatic complementarity with the binding pocket. Then, we evaluated the optimal concentration of the candidate drugs and found that they not only significantly inhibited the proliferation of HCC cells but also inhibited the protein level of YAP1, partly reflecting the inhibitory effect of SMARCAL1 (Fig. 9B-C). However, the role of SMARCAL1 inhibitor is only preliminary exploration, and a large number of experiments and possible clinical trials are still needed to confirm its potential anti-tumor value. All in all, these results suggested that the combination of SMARCAL1-targeting inhibitors with existing anti-tumor agents that act on the Hippo pathway might represent a novel strategy for addressing liver cancer in the future.

## Discussion

The onset of liver cancer is insidious, often leading to diagnosis in the middle to late stages, which contributes to its high mortality rate^[33]^. Although drug therapy has progressed significantly in HCC patients, it is not generally effective^[34]^. Therefore, finding early specific markers of liver cancer is warranted. Here, we identified SMARCAL1 as an innovative prognostic factor for liver cancer development. Primarily, we explored the expression patterns and clinical properties of SMARCAL1 from TCGA and GEO databases and found that SMARCAL1 was specifically highly expressed in HCC, other than hepatitis, and liver cirrhosis. Prognostic analysis showed that SMARCAL1 could be an independent prognostic factor, and its high expression was closely related to the poor prognosis of HCC. Then, we also validated the proliferation ability of SMARCAL1 in liver cancer; the results indicated that knockdown of SMARCAL1 can significantly inhibit the growth of liver cancer cell, consistent with the results of TCGA analysis. Notably, SMARCAL1 expression was also differentially expressed in HCC stage; more importantly, survival analysis of patients with T1 + T2, I + II, N0, and M0, respectively, showed high expression of SMARCAL1 indicating a worse prognosis at the early stage of liver cancer. These findings suggested that the high expression of SMARCAL1 may be related to the susceptibility to the development of advanced HCC, and it is expected to be a marker for early screening of HCC.

The microenvironment of liver cancer is prone to forming an immunosuppressive microenvironment due to heterogeneity and genetic instability, thereby developing tolerance to immunosuppressants^[35]^. Tumor-associated macrophages (TAMs), as the most abundant inflammatory cells in the TME, are highly correlated with the poor prognosis and immunotherapy response of patients with various cancers^[36]^. In our study, we have found that high expression of SMARCAL1 was positively with macrophages, especially the M2 subtype, additionally, patients with SMARCAL1-high combined M2 macrophages-high showed poorer prognosis than patients with SMARCAL1-high/M2 macrophages-low. Meanwhile, TIDE score illustrated that high expression of SMARCAL1 made it more difficult to benefit from immunosuppressants. These results may explain the poor efficacy of immunosuppressants in patients with liver cancer and possibly targeting SMARCAL1, which may improve the resistant immune microenvironment of liver cancer by regulating macrophage differentiation, has become an emerging strategy.

Regulating the Hippo signaling pathway, especially inhibiting the expression of YAP and cellular sub-localization, has become an effective potential target for the treatment of primary liver cancer^[37]^. Here, we found that SMARCAL1 and YAP had spatial co-localization, and molecular docking also demonstrated a strong interaction between them, suggesting that SMARCAL1 may regulate the transcriptional activity or subcellular localization of YAP through direct physical interaction, thereby establishing a functional link between SMARCAL1 and the Hippo signaling pathway. Additionally, the detailed regulatory mechanism of SMARCAL1 on Hippo pathway was further validated at the protein and gene levels. YAP can promote the secretion of some chemokines by tumor cells, attract regulatory T cells (Tregs) to aggregate in tumor tissues, and promote the polarization of macrophages toward type M2^[38,39]^. Interestingly, we also discovered that SMARCAL1 was positively correlated with Tregs and M2 macrophages. Whether SMARCAL1 induces the formation of a resistant immune microenvironment in HCC by regulating the activity of YAP and whether targeting SMARCAL1 can promote the transformation of HCC from a “cold tumor” to a “hot tumor” remain to be investigated.

Drugs that target SMARCAL1 are currently being explored to enhance the efficacy of immunotherapy by interfering with its dual role in cancer immune escape. Pixantrone, as a newly discovered SMARCAL1 small molecule inhibitor in SCLC patients, received good results in the Phase I clinical trial^[13]^. Although our research also identified two candidate small molecule inhibitors of SAMRCAL1 activity and initially demonstrated their anti-proliferative ability and regulation of downstream proteins in liver cancer cells, this is still in the initial exploration stage. *In vivo* validation and future clinical implementation require a significant amount of work and time to complete. Future therapies that focus on SMARCAL1 could pave the way for more precise immune system regulation, potentially enhancing the efficacy of cancer treatments, particularly for patients who show limited response to current therapies. Notably, due to the complex immune microenvironment of liver cancer, inhibitors targeting SMARCAL1 will pose greater challenges, such as uncertainty of dosage, the risk of side effects, and difficulties in clinical transformation, which is also the direction that we will continue to break through.

## Data Availability

The public datasets generated or analyzed during the present study are available in the TCGA database (https://portal.gdc.cancer.gov/). The data used in this study are available from the corresponding authors.

## Research registration unique identifying number (UIN)

Not applicable. Our research does not involve clinical studies and does not require submission for identifying number of study registration.
